# A Prospective Open‐Label Study of Tolerance and Effectiveness of Sequential Dermocosmetic Treatments Combining Poly‐l‐Lysine Biovectors With Vitamins A and C

**DOI:** 10.1002/hsr2.70676

**Published:** 2025-04-16

**Authors:** Bertrand Nassar, Hafid Belhadj‐Tahar, Weiyang Jin, Guanghua Yang

**Affiliations:** ^1^ Research and Expertise Group, French Association for Medical Research Advancement (AFPREMED) Toulouse France; ^2^ Research Department Dermabel Cosmetics France Toulouse France; ^3^ International Research Center for Biological Sciences Shanghai Ocean University Shanghai China

**Keywords:** atopic dermatitis, dermocosmetics, eczema, poly‐l‐lysine, pruritus, psoriasis

## Abstract

**Background and Objectives:**

Atopic dermatitis (AD) is a common chronic inflammatory skin condition, affecting 15%–20% of children and up to 10% of adults. It has a significant impact on patients' quality of life and represents a considerable burden on healthcare systems. Management strategies aim to restore the skin barrier, reduce inflammation, and achieve physiological and homeostatic balance. This study evaluates the safety and efficacy of a new dermocosmetic formulation combining poly‐l‐lysine biovectors with vitamins A and C.

**Methods:**

A single‐arm, open‐label study was conducted on 20 patients diagnosed with AD, including 14 women and 6 men aged between 24 and 63, with skin phototypes of Fitzpatrick I–VI. Treatment involved sequential application of poly‐l‐lysine G2/vitamin C in the morning and poly‐l‐lysine G3/vitamin A in the evening for 28 days, on the face, hands, and body. Outcomes were assessed using the Dermatology Life Quality Index (DLQI) and SCORAD scores. Additional assessments included a visual analog scale for pruritus and sleep disturbance, as well as patient self‐report questionnaires on skin improvement and treatment satisfaction.

**Results:**

The treatment was well‐tolerated, with no adverse effects and no relapses of AD. After 28 days, patients showed a significant reduction in DLQI (−61.8%, Cohen's *d* = 1.20) and SCORAD (−41.8%, Cohen's *d* = 1.05) scores. Objective improvements included reductions in erythema (−50%), dryness (−60%), and pruritus (−50%).

**Conclusion:**

This study demonstrates the potential of poly‐l‐lysine‐based dermocosmetics combined with vitamins A and C to improve the symptoms and quality of life of patients suffering from AD. The formulations were well‐tolerated and effective in restoring the skin barrier and reducing inflammation. Further large‐scale controlled studies are needed to validate these results and explore long‐term efficacy.

## 1 Introduction

Atopic dermatitis (AD) is a chronic, disabling inflammatory skin condition that mainly affects children, but can also be present in adults [1]. AD is a major health problem worldwide, with a prevalence of 15%–30% in children and 2%–10% in adult [2]. In industrial countries, the prevalence of AD has increased two to threefold over the last 30 years [3]. AD is characterized by symptoms such as itchy, red, dry, and inflamed skin. Although there is no definitive cure, the disease can be well managed with the use of emollients, topical corticosteroids (TCSs), and oral treatments such as antihistamines and immunosuppressants.

AD has been shown to have an important impact on quality of life in children and adults, and its chronic and relapsing course represents an ongoing problem in their daily [4, 5, 6, 7]. AD is implicated in psychological disorders linked to altered body schema and contributes to social isolation, negative social comparisons, negative self‐image, stigma, and social rejection [8].

The pathogenesis of AD is incompletely understood but involves a dysregulation of inflammation and the inappropriate response to antigens [9, 10, 11]. Therapy for AD has largely focused on agents that act on the dysfunctional inflammatory response, including anti‐inflammatory and immunosuppressive agents. In this context, the spectrum of topical therapies used to treat AD ranges from emollients to potent anti‐inflammatory and immunomodulatory agents, including TCSs, and topical calcineurin inhibitors (TCIs), more recently developed compounds with a more selective effect on immunoregulation [12]. These topical treatments for AD, while effective in controlling disease activity and maintaining clinical remission, can also induce local adverse effects, including infections and skin atrophy [13, 14, 15, 16]. Similarly, the use of these powerful anti‐inflammatory and immunomodulatory drugs can lead to systemic side effects that are detrimental to the health of the individual. These effects depend on the duration of treatment and the surface area of the body treated [17].

As AD is a chronic skin disease, the skincare sector has recently had to adapt to respond rapidly to new needs and demands for dermocosmetics as alternatives to the classic treatment of AD [18, 19]. The greatest challenge is to develop new products that are effective, harmless to health and the environment, and part of a holistic vision that considers the well‐being of both the individual and the planet. There is currently an unmet need for skincare products for atopic skin (eczema, psoriasis, etc.) formulated with natural active ingredients derived from plants or produced in the laboratory using green chemistry processes [20].

In this context, combining vitamin A with vitamin C appears to be an optimal solution. Vitamins C and A have a complementary action for healthy skin and a synergistic action (anti‐inflammatory/healing) for the treatment of these skin pathologies. Vitamin C, being a powerful reducing agent, can regulate the resolution of the inflammatory process and stimulate tissue repair. It modulates the release of catabolic inflammatory cytokines, chemotaxis of immune cells and activation of phagocytosis [21, 22, 23]. Vitamin A exerts a regulatory action in particular on Langerhans cells, which have receptors on their surface with numerous functions in the immune response (phagocytosis of immune complexes and antibody‐dependent cell‐mediated cytotoxicity (ADCC)), cell proliferation, differentiation, and keratinization. Langerhans cells also play an important role in certain dermatoses with a rapid mitotic index, such as psoriasis. The involvement of Langerhans cells has been demonstrated in the majority of inflammatory skin diseases such as allergic contact dermatitis, AD, and psoriasis [24]. Furthermore, as emphasized by Dharshan and colleagues, vitamin D3 and dioxopiperidinamide derivatives demonstrate significant anti‐inflammatory and antioxidant properties, suggesting their potential for therapeutic applications [25]. The effectiveness of the products depends on the stability of vitamins and derivatives in contact with oxygen and light, and their penetration through the skin to reach their sites of action.

As a result, formulations are increasingly based on a delivery system using vectors to optimize efficacy by modulating the release and absorption of the active ingredient. In cosmetics, the main concern is to reach skin targets while limiting passage into the bloodstream. The delivery system offers an interesting approach to the administration of molecules of therapeutic interest by conveying them using a vector in the form of a vesicle, particle, or emulsion [26]. Moreover, encapsulation technologies have attracted considerable attention for their ability to protect sensitive compounds from oxidative degradation, improve stability, and enhance targeted delivery. Nanoencapsulation of bioactive compounds, such as vitamins, minerals, and essential fatty acids, has shown remarkable potential to increase bioavailability and targeted efficacy, particularly in the food and pharmaceutical industries [27].

New research on polylysine‐based drug delivery systems highlights its multifunctional properties, including pH responsiveness, enzyme resistance, and the ability to enhance cellular uptake and stability. These features position polylysine as an outstanding candidate for advanced delivery platforms in biomedical and cosmetic applications [28].

In this context, we have developed formulations based on native vitamins coupled to a vector made exclusively of poly‐amino acids (poly‐l‐lysines) obtained by green chemistry, which is biocompatible, biodegradable, and harmless to the skin. In addition, mastery of the synthesis of this poly‐l‐lysine means that vectors of varying sizes and physicochemical properties can be obtained [29, 30, 31].

We report here the results of safety and efficacy against AD of a new cosmeceuticals formulation combining vitamins A and C with a second‐ and third‐generation poly‐l‐lysine during 4 weeks.

## 2 Methods

This prospective, single‐arm (uncontrolled), open‐label study comprised a limited number of 20 patients with AD, including 14 women and 6 men aged 24–63 years. The diagnosis was established according to Hanifin and Rajka [32]. The inclusion and exclusion criteria are set out in the box below (Table 1).

**Table 1:** The inclusion and exclusion criteria.

Inclusion criteria
1. Males or Females between 18 and 60 years (*N* = 25) have skin that is dry/atopic or very dry/atopic (confirmed by the Dermatologist) 2. have mild atopic eczema (as confirmed by the Dermatologist in consultation) but not undergoing steroid treatment for the condition 3. Treatment area amenable to topical treatment 4. Following verbal and written information about the trial, the patient must provide signed and dated informed consent before any study‐related activity is carried out, including activities relating to washout period

Exclusion criteria
1. have chronic or acute skin diseases, except atopic eczema or psoriasis with a maximum PGA score of 2 on any part of the body 2. have an active flare up of atopic eczema on any sites of the body at screening 3. known allergy or sensitivity to cosmetic products and/or any ingredients of the investigational product 4. any systemic illness that would impact on the subjects safety or wellbeing and/or affect the response of the skin or the interpretation of the test results at screening (Days −14 to −7) 5. receiving the following topical or systemic treatments at baseline (Day 0); o anti‐inflammatory and/or antihistamines during the previous week o cough suppressants and/or topical or inhaled corticosteroids during the previous 2 weeks o retinoids and/or immunosuppressants during the previous 6 months 6. participation in other studies/investigations on any part of the body during the last 4 weeks 7. use of dermatological therapeutics on the body within 7 days prior to baseline (Day 0) (use of such products on the face, trunk, hands, and nappy area is permitted) 8. intensive/prolonged exposure to the sun within 30 days prior to screening 9. subjects in protective care 10. Any clinically significant and relevant abnormalities of medical history and/or any condition or medication that would impact on the subject safety or well‐being and/or affect the response of the skin or the interpretation of the test results in the opinion of the Principal Investigator or designee

The study was conducted under dermatological supervision in an accredited facility and in compliance with Good Clinical Practice (GCP) guidelines. The study adhered to the principles outlined in the Declaration of Helsinki, and informed consent was obtained from all participants prior to the study.

Participants received treatment with poly‐l‐lysine vitamin dermocosmetic preparations (from Dermabel Cosmetics France Laboratories, Toulouse, France) for topical application on the entire body twice daily for 4 weeks. The poly‐l‐lysine G2/vitamin C formulation is applied at least once in the morning while poly‐l‐lysine G3/vitamin A in the evening.

The Dermatology Life Quality Index (DLQI) was used to assess the impact of AD on the lives of the patients [33].

A visual analog scale was used to assess pruritus and sleep disturbance, where 0 indicates no pruritus/sleep disturbance and 10 the most extreme form. The investigating dermatologist calculated the SCORAD index (a clinical tool for assessing the severity, i.e., extent and intensity, of AD). Assessments of pruritus, DLQI, and SCORAD were carried out twice before (baseline assessment) and at the end of the study [34]. Effectiveness self‐assessment questionnaires: immediately after the first application of vitamin C (in the morning) and vitamin A (in the evening) (closed questions with a 4‐point semantic scale). On the 28th day of the test (closed questions with a 4‐point semantic scale). Each response rated from 0 to 4 (*Strongly Agree*: 1, *Agree*: 2, *Disagree*: 3, *Strongly Disagree*: 4) is considered positive if its value is > 2.

The statistical analyses were performed using Microsoft Excel 2021. Descriptive statistics (mean ± standard deviation) were used to summarize baseline characteristics and outcomes. Paired Student's *t*‐tests were applied to evaluate within‐group changes from baseline to the end of the study. Statistical significance was defined as *p* < 0.05, and all tests were two‐sided. For nonparametric data, Wilcoxon signed‐rank tests were considered. Effect sizes (Cohen's *d*) were calculated for primary outcomes (DLQI and SCORAD) to measure the magnitude of treatment effects. Exploratory analyses were conducted to assess patient‐reported outcomes and differences between subgroups based on comorbidities (e.g., psoriasis and allergic rhinitis).

## 3 Results

This prospective, single‐arm (uncontrolled), open‐label study comprised a limited number of 20 patients with AD, including 14 women and 6 men aged 24–63, with a mean age of 43.6 ± 11.5 years and Fitzpatrick skin phototypes I–VI, as detailed in Table 1. Of the 20 patients, 9 had known allergies and 5 suffered from asthma or allergic rhinitis, as shown in Table 2.

**Table 2:** Epidemiological and clinical characteristics of the volunteers.

Subject	Sex	Age	Phototype	Skin pathology	Allergenic agent	Comorbidity
#1	F	45	II	AD	Nickel	
#2	F	43	VI	AD	Beta lactam	Asthma
#3	M	48	III	AD	Pollen	Allergic rhinitis
#4	M	63	II	AD		
#5	F	24	II	AD	Nut	
#6	F	33	II	AD		
#7	M	37	III	AD	Pollen	
#8	M	48	II	AD, Psoriasis		
#9	F	41	II	AD		
#10	F	62	II	AD		Allergic rhinitis
#11	F	34	II	AD	Pollen	
#12	F	57	II	AD		
#13	F	61	III	AD		
#14	F	54	III	AD		
#15	M	48	II	AD		Asthma
#16	F	27	VI	AD	Pollen, Mites	Asthma
#17	F	27	IV	AD	Nickel	
#18	F	45	III	AD		
#19	F	36	VI	AD		
#20	M	40	VI	AD, Psoriasis	Pollen, Mites	

After 28 days of treatment, Table 3 shows a significant reduction in SCORAD (−41.8%, Cohen's *d* = 1.05) scores, while Table 4 highlights a significant reduction in DLQI (−61.8%, Cohen's *d* = 1.20), indicating large effect sizes for both primary outcomes. These findings underscore the clinical relevance of the treatment effects. More precisely, Table 3 emphasizes the secondary outcomes, including reductions in erythema (−50%) and dryness (−60%), which also demonstrated significant improvements with moderate to large effect sizes (Cohen's *d* > 0.8).

**Table 3:** Comparative SCORAD score at the start (Day 1) and end of the study (Day 28).

	Intensity items						Subjective items	
	Erythema	**Œdema**	Oozing ⁄crust	Effect of scratching	Lichenification	Dryness	Sleeplessness	Itch
#1	1 (0)	0 (0)	0 (0)	2 (0)	2 (1)	2 (1)	0 (0)	6 (1)
#2	0 (0)	1 (0)	0 (0)	0 (0)	0 (0)	1 (0)	0 (0)	9 (0)
#3	2 (1)	1 (2)	1 (1)	0 (1)	0 (0)	1 (1)	0 (0)	3 (6)
#4	2 (0)	0 (1)	1 (1)	1 (0)	0 (0)	0 (0)	0 (0)	4 (0)
#5	1 (3)	1 (2)	0 (1)	0 (0)	0 (0)	0 (0)	0 (6)	3 (6)
#6	2 (0)	0 (1)	0 (0)	0 (0)	0 (1)	2 (1)	5 (2)	6 (3)
#7	2 (4)	0 (1)	0 (0)	0 (0)	0 (0)	1 (0)	0 (0)	6 (6)
#8	1 (1)	0 (0)	0 (0)	0 (1)	0 (0)	2 (0)	0 (0)	1 (0)
#9	1 (0)	0 (1)	1 (1)	0 (0)	1 (1)	1 (1)	0 (0)	7 (0)
#10	1 (0)	0 (0)	0 (0)	0 (0)	0 (0)	2 (1)	0 (0)	7 (0)
#11	0 (0)	1 (0)	0 (0)	0 (1)	0 (0)	2 (1)	0 (6)	6 (6)
#12	2 (0)	0 (0)	1 (0)	0 (0)	0 (0)	2 (2)	5 (0)	1 (0)
#13	1 (0)	0 (0)	0 (0)	0 (0)	0 (0)	0 (0)	0 (0)	4 (0)
#14	1 (0)	1 (1)	0 (0)	0 (0)	0 (0)	2 (1)	0 (0)	3 (3)
#15	2 (1)	0 (0)	0 (0)	0 (0)	1 (0)	0 (0)	9 (0)	8 (2)
#16	0 (0)	1 (0)	0 (0)	0 (0)	1 (0)	1 (0)	4 (0)	5 (3)
#17	1 (0)	0 (1)	0 (0)	0 (0)	0 (0)	1 (0)	0 (0)	1 (4)
#18	0 (0)	1 (0)	0 (0)	0 (0)	0 (0)	1 (0)	0 (0)	3 (0)
#19	0 (0)	1 (0)	0 (0)	0 (0)	2 (1)	1 (0)	5 (0)	7 (3)
#20	0 (0)	0 (0)	0 (0)	0 (0)	2 (2)	2 (1)	2 (0)	0 (2)
Total	20 (10)	8 (10)	4 (4)	3 (4)	9 (6)	24 (30)	30 (14)	90 (45)
Moyenne	1 (0.5)	0.4 (0.5)	0.2 (0.2)	0.15	0.45 (0.3)	1.2 (0.5)	1.5 (0.7)	4.5 (2.25)
Min/Max	0–2 (0–2)	0–2 (0–2)	0–1 (0–1)	0–2	0–2 (0–2)	0–2	0–9 (0–6)	0–8 (0–6)
*t* student (*p*)	0.007	NS	NS	NS	NS	0.003	NS	0.006

*Note:* Value (*x*) represents the score obtained at the end of the study.

Abbreviation: NS, no significant.

**Table 4:** Comparative DLQI score at the start (Day 1) and end of the study (Day 28).

	Baseline	Week 4
	Mean (± SD)	Mean (± SD)
DLQI score	7.6 (4.8)	2.9 (2.8)
% reduction change in DLQI scores	61.8	
*p* value (vs. baseline)	0.001	
Score maximum	23	14
Score minimum	3	0

The treatment was generally well‐tolerated, with only two minor and transient adverse events reported, which were not found at the end‐of‐study consultation, as shown in Table 3: Subject #8, who experienced a sensation of heat in the treated areas on Days 2 and 3, and Subject #17, who presented slight desquamation in the treated facial area during Weeks 2 and 3.

Self‐assessment of efficacy indicates that the application of vitamin C from the first use improves all subjective and objective signs, particularly scratching and redness, and has a soothing effect. As shown in Table 5, on the first day of treatment, there was no significant difference between the application of the vitamin C formula alone and the sequential combination of vitamin C applied in the morning and vitamin A in the evening. However, Table 5 also demonstrates that, at the end of the study, the sequential combination of vitamin C with vitamin A significantly reduced redness and irritation while improving skin quality and promoting the repair of damaged skin.

**Table 5:** Comparative self‐assessment score at the start (Day 1) and end of the study (Day 28).

Item	Day 1		Day 28
	Vitamin C (*n*/20) %	Vitamin A (*n*/20) %	Association vitamins A & C (*n*/20) %
The skin appears more supple	(16/20); 81%	(16/20); 81%	(18/20); 90%
The routine reduces skin redness	(10/20); 48%	(10/20); 52%	(16/20); 90%
The skin appears more nourished	(15/20); 76%	(16/20); 81%	(17/20); 85%
The skin appears more hydrated	(18/20); 90%	(16/20); 81%	(16/20); 80%
The product/routine reduces skin tightness	(17/20); 86%	(17/20); 86%	(16/20); 80%
The skin appears less damaged	—	—	(16/20); 80%
The skin seems more protected	(14/20); 71%	(14/20); 71%	(18/20); 80%
The skin appears softer	(14/20); 71%	(13/20); 67%	(15/20); 75%
The skin appears more soothed	(14/20); 62%	(14/20); 71%	(16/20); 75%
The skin appears more repaired	—	—	(16/20); 75%
The routine reduces skin irritation	—	—	(16/20); 75%
Since using this routine, I have noticed an improvement in the quality of my skin	—	—	(16/20); 75%
The skin seems more comfortable	(18/20); 90%	(17/20); 86%	(14/20); 70%
Lesions/plaques on my skin seem less visible	—	—	(14/20); 70%
The routine helps reduce/limit the appearance of new lesions/plaques	—	—	(14/20); 70%
The skin seems more protected from external aggressions	—	—	(14/20); 70%
The routine reduces itchy skin	(18/20); 71%	(18/20); 90%	(13/20); 65%
Since using this routine, I have noticed a reduction in my skin problems	—	—	(13/20); 65%

For patients #8 and #20 suffering from psoriasis associated with AD, as mentioned in Table 2, their average PASI score fell by 46.7%: from an initial average score of 0.8 to 0.4 at the end of the study.

## 4 Discussion

AD is the most common chronic inflammatory skin condition, with a prevalence of 15%–20% in children and up to 10% in adults [1, 35].

AD not only affects the patient's health and quality of life, but also represents a major burden on the healthcare system [36].

The etiology of AD is multifactorial: dysfunction of the immune system, alteration of the skin barrier, genetic and environmental factors [37, 38, 39]. Although the interaction between these factors is not fully understood, it appears that the combined and synergistic action of all these factors leads to a physiological alteration of the skin barrier. The skin becomes dry, irritated, erythematous, exudative, and prone to infections. Some lesions, after intensive scratching, become lichenified.

The objective of the management of AD is to treat the defect of the skin barrier and the inflammation and to restore its physiological and homeostatic balance, thus obtaining a prolonged remission of the patient. Topical therapies play a key role in achieving these goals [40].

The range of topical therapies used to treat AD varies from emollients to potent anti‐inflammatory and immunomodulatory agents, such as TCSs and TCIs, recently developed compounds with a more selective effect on immune regulation. For treatment, the correction of barrier defect is the first practical step before specific therapies such as TCSs, and/or TCIs. Although these topical treatments TCs and TCIs are effective in controlling disease activity and maintaining clinical remission, they can also cause local adverse effects, including infections and skin atrophy. Similarly, the use of these powerful anti‐inflammatory and immunomodulatory drugs can lead to systemic side effects that are detrimental to an individual's health. These side effects depend on the duration of treatment and the body surface area treated [17].

Since AD is a chronic skin disease that particularly affects young children, the greatest challenge is to develop new dermocosmetics that are effective and harmless to health and the environment, for everyday skin care.

In this context, combining vitamin A with vitamin C appears to be an optimal solution. Vitamins C and A have a complementary action for healthy skin and a synergistic action (anti‐inflammatory/healing) for the treatment of these skin pathologies. The effectiveness of the products depends on the stability of these two vitamins in contact with oxygen and light, as well as their transcutaneous penetration to reach their sites of action.

In cosmetics field, the main concern is to reach skin targets while limiting passage into the bloodstream. The delivery system offers an interesting approach for administering molecules of therapeutic interest by carrying them using a vector in the form of a vesicle, particle or emulsion [25]. With this in mind, we have developed formulations based on native vitamins coupled with a carrier consisting exclusively of poly‐amino acids (poly‐l‐lysines) which are biocompatible, biodegradable, and harmless to the skin. In addition, the combination of these native vitamins with the poly‐l‐lysine dendrimer ensures the stability of these fragile vitamins in the presence of oxygen and light, as well as their transcutaneous delivery [29, 30, 31].

In the present study, 20 patients with AD, aged between 24 and 64 years, and with a skin phototype of Fitzpatrick I–VI, were included. Of the 20 patients, 9 had known allergies, 5 had asthma or allergic rhinitis, and 2 had psoriasis associated with AD (Table 2).

The clinical characteristics align with the diagnostic standards established for AD and the comorbidities reported by Maintz and colleagues highlighting the systemic impact of AD [18, 32].

The skin treatment consisted of a sequential combination of applications of a poly‐l‐lysin/vitamin C‐based formula to the face, hands, and body during the day and a poly‐l‐lysin/vitamin A‐based formula in the evening. The treatment was generally well‐tolerated, no AD flare‐ups were observed during the 28‐day treatment period (Tables 3 and 5). This aligns with previous studies, such as Lavanya and colleagues, which demonstrated that encapsulation of active ingredients, like those used in this study, improves tolerance by minimizing direct irritation and enhancing the stability and targeted delivery of sensitive compounds [27].

In addition, there was a significant improvement in the individual's quality of life thanks to the efficacy of this combination on the intensity of objective and subjective signs, as shown by the change in the SCORAD score (−41.8%) and DLQI score (−61.8%) (Tables 3 and 4). Treatment with vitamins A and C resulted in a significant reduction (*p* < 0.01) in erythema (−50%), dryness (−60%), and pruritus (−50%) (Table 3). These findings are consistent with previous studies, which demonstrated that vitamins A and C improve skin disorders by reducing inflammation and pruritus while strengthening the skin barrier, thereby enhancing both clinical outcomes and patient‐reported satisfaction [41, 42].

Self‐assessment of effectiveness shows that on the first day of treatment, the effect of the vitamin C applied seems to immediately improve all subjective and objective signs, in particular scratching and redness, and has a soothing effect, whereas the application of the vitamin A formulation following the night seems to contribute little.

However, at the end of the study, the sequential combination of poly‐l‐lysin/vitamin C and poly‐l‐lysin/vitamin A significantly reduced both redness and irritation, while improving skin quality and repairing damaged skin over the long term and preventing the occurrence of new flare‐ups. Previous studies have demonstrated the immediate action of vitamin C with poly‐l‐lysine, linked to its topical anti‐free radical and anti‐inflammatory action (soothing effect), while vitamin A has a delayed action, repairing damaged skin and restoring the skin's physiological balance in synergy with vitamin C poly‐l‐lysine [29].

In fact, the vitamins and their metabolites play a determining role depending on time and dose, on the proliferation and differentiation of epithelial cells, epithelial and mesenchymal synthetic performance, immune modulation as well as protection. against cancer and other diseases, including autoimmune and infectious diseases [21, 22, 23].

Vitamins and their derivatives and in particular A (retinol and its derivatives), C (ascorbic acid and its derivatives), and E (tocopherol) are commonly used in topical formulations, due to the biological effects linked to their antiaging and antioxidant properties and anti‐inflammatories.

However, several of these formulations are not sufficiently effective in cutaneous application due to a very low concentration of these vitamins delivered in relation to their low bioavailability due to their virtual nonpenetration into the skin tissue.

In order to overcome the disadvantages, the two formulations have been proposed combining vitamin A and vitamin C with the poly‐l‐lysine vector. This choice is based on the complementary action of these two vitamins for healthy skin and their synergistic action for the treatment of skin pathologies.

‐ On the one hand, the antiradical and anti‐inflammatory action of vitamin C:

Vitamin C (Vit C) or ascorbic acid is a hydrophilic molecule that has essential physiological and metabolic activities in humans, it reduces unstable species of oxygen, nitrogen, and sulfur radicals and helps regenerate other antioxidants in the body, such as alpha‐tocopherol (vitamin E). Vitamin C has an antiradical action, it deactivates free radicals induced by ultraviolet radiation, but also other physical or chemical agents causing skin burns; it has an anti‐inflammatory action [21].

Vitamin C, being a powerful reducing agent, can regulate the resolution of the inflammatory process and stimulate tissue repair. It modulates the release of catabolic inflammatory cytokines, the chemotaxis of immune cells and the activation of phagocytosis [23].

Furthermore, vitamin C is the essential cofactor of the two enzymes necessary for the synthesis of collagen: prolyl hydroxylase and lysyl hydroxylase. In addition, it has an inhibitory action on metalloproteinase‐I (MMP‐I), which thus reduces the degradation of collagen.

‐ On the other hand, cellular and tissue actions of vitamin A:

Vitamin A exerts a regulatory action in particular on Langerhans cells which present on their surface receptors which have numerous functions in the immune response (phagocytosis of immune complexes and ADCC), in the cellular proliferation, differentiation, and keratinization. They have phagocytic and enzymatic properties and are responsible for capturing allergens to present them to T lymphocytes. They thus trigger a cellular immune response which is responsible for Type IV contact allergy or delayed hypersensitivity. In addition, these Langerhans cells play an important role in certain dermatoses with a rapid mitotic index such as psoriasis. The involvement of Langherans cells has been demonstrated in the majority of inflammatory skin pathologies such as allergic contact dermatitis, AD, and psoriasis [24].

## 5 Conclusion

AD is the most common chronic inflammatory skin condition. Its management aims to treat the skin barrier defect and inflammation and restore its physiological and homeostatic balance, in order to achieve a prolonged remission in the patient. Topical therapies play a key role in achieving these objectives. This prospective study highlights the safety and efficacy of a 28‐day sequential treatment based on formulations combining poly‐l‐lysin and vitamins A and C to treat AD. The results show a significant improvement in patients' quality of life and a reduction in symptoms such as erythema, dryness, and pruritus. These results reinforce the promising role of dermocosmetics incorporating biocompatible vectors to stabilize and carry sensitive active ingredients such as vitamins.

However, challenges remain, including exploring the long‐term efficacy of these formulations and their performance in larger, more diverse patient groups. Understanding the complex interactions between active ingredients and skin barriers, and optimizing them for different populations and phototypes, also represents a crucial avenue for future research.

In the future, the focus will be on developing controlled multicenter studies to confirm these preliminary results and extend their clinical application. In addition, work on the sustainability of production processes and their environmental impact is needed to align these innovations with an eco‐responsible and holistic approach. These prospects promise to position dermocosmetics as viable and effective solutions for the management of chronic skin pathologies.

## Author Contributions

**Bertrand Nassar:** investigation, methodology. **Hafid Belhadj‐Tahar:** conceptualization, methodology, supervision, writing – review and editing. **Weiyang Jin:** data curation, methodology, writing – review and editing. **Guanghua Yang:** formal analysis, investigation. All authors were involved in drafting, revising, and approving the final version of the manuscript.

## Conflicts of Interest

The authors declare no conflicts of interest.

## Transparency Statement

The lead author Hafid Belhadj‐Tahar affirms that this manuscript is an honest, accurate, and transparent account of the study being reported; that no important aspects of the study have been omitted; and that any discrepancies from the study as planned (and, if relevant, registered) have been explained.

## Data Availability

The authors confirm that the data supporting the findings of this study are available within the article and no supporting files have been submitted.
